# CD4^+^ and CD8^+^ T Cells Exert Regulatory Properties During Experimental Acute Aristolochic Acid Nephropathy

**DOI:** 10.1038/s41598-018-23565-2

**Published:** 2018-03-28

**Authors:** Thomas Baudoux, Cécile Husson, Eric De Prez, Inès Jadot, Marie-Hélène Antoine, Joëlle L. Nortier, Jean-Michel Hougardy

**Affiliations:** 1 Laboratory of Experimental Nephrology, Faculty of Medicine, Université libre de Bruxelles, Brussels, Belgium; 2 Service of Nephrology, Dialysis and Renal Transplantation, Erasme Hospital, Université Libre de Bruxelles, Brussels, Belgium; 3 Molecular Physiology Research Unit — URPhyM, NARILIS (Namur Research Institute for Life Sciences), University of Namur (UNamur), Namur, Belgium

## Abstract

Experimental aristolochic acid nephropathy is characterized by transient acute proximal tubule necrosis and inflammatory cell infiltrates followed by interstitial fibrosis and tubular atrophy. The respective role of T-cell subpopulations has never been studied in the acute phase of the mouse model, and was heretofore exclusively investigated by the use of several depletion protocols. As compared to mice injected with aristolochic acids alone, more severe acute kidney injury was observed after CD4^+^ or CD8^+^ T-cells depletion. TNF-alpha and MCP-1 mRNA renal expressions were also increased. In contrast, regulatory T-cells depletion did not modify the severity of the aristolochic acids induced acute kidney injury, suggesting an independent mechanism. Aristolochic acids nephropathy was also associated with an increased proportion of myeloid CD11b^high^F4/80^mid^ and a decreased proportion of their counterpart CD11b^low^F4/80^high^ population. After CD4^+^ T-cell depletion the increase in the CD11b^high^F4/80^mid^ population was even higher whereas the decrease in the CD11b^low^F4/80^high^ population was more marked after CD8+ T cells depletion. Our results suggest that CD4^+^ and CD8^+^ T-cells provide protection against AA-induced acute tubular necrosis. Interestingly, T-cell depletion was associated with an imbalance of the CD11b^high^F4/80^mid^ and CD11b^low^F4/80^high^ populations.

## Introduction

Human aristolochic acid nephropathy (AAN) was formerly known as “Chinese herb nephropathy”. This tubulointerstitial nephritis was first reported in Belgian women after ingestion of herbal slimming remedies containing aristolochic acids (AA). These nitrophenanthrene derivatives were found responsible for the so-called Balkan endemic nephropathy and for hundreds of cases of chronic renal failure in China and Taiwan where traditional herbal medicines are still widely used^1–3^. Histologically, AAN displays an interstitial fibrosis with a typical corticomedullary gradient and tubular atrophy^4,5^. AAN was reproduced in several animal models including rabbits, mice and rats^6–9^. Prior time courses studies of our Wistar rat model demonstrated a biphasic evolution of tubulo-interstitial lesions^10,11^. During the *acute phase*, a necrosis of proximal tubular epithelial cells (PTEC) followed by a macrophage, CD4^+^ and CD8^+^ T-cell infiltration were observed. Thereafter, interstitial fibrosis and tubular atrophy were the hallmark of the *chronic phase*. In this model, inflammatory infiltrate (macrophages (mϕ) followed by CD4^+^ and CD8^+^ T-cells) were proposed as the physiopathological link between the *acute* and the *chronic phases*^10^. Apart from AAN, the role of the immune system has been largely studied in both ischemic and other toxic acute kidney injury (AKI) models. In response to acute tubular necrosis (ATN), inflammatory cytokines produced by PTEC, resident and infiltrating leukocytes contribute to the accumulation of inflammatory cells into the interstitium^12^. The roles of various immune cells, including dendritic cells (DC), natural killer T-cells (NKT), T and B lymphocytes, neutrophils and mϕ have been described^12^, all of which ultimately lead to tubular atrophy and interstitial fibrosis. However, the respective role of each of these cell types remains controversial since the observed data depends on the type of model used. Moreover, the role of these different T-cell types has never been studied in experimental AAN. We therefore decided to investigate the potential role of CD4^+^ and CD8^+^ T-cells during the acute phase of toxic nephropathy, by studying the effects of selective depletion using monoclonal antibodies.

## Results

### CD4^+^ depletion aggravates acute kidney injury in experimental AAN

**Figure 1:**
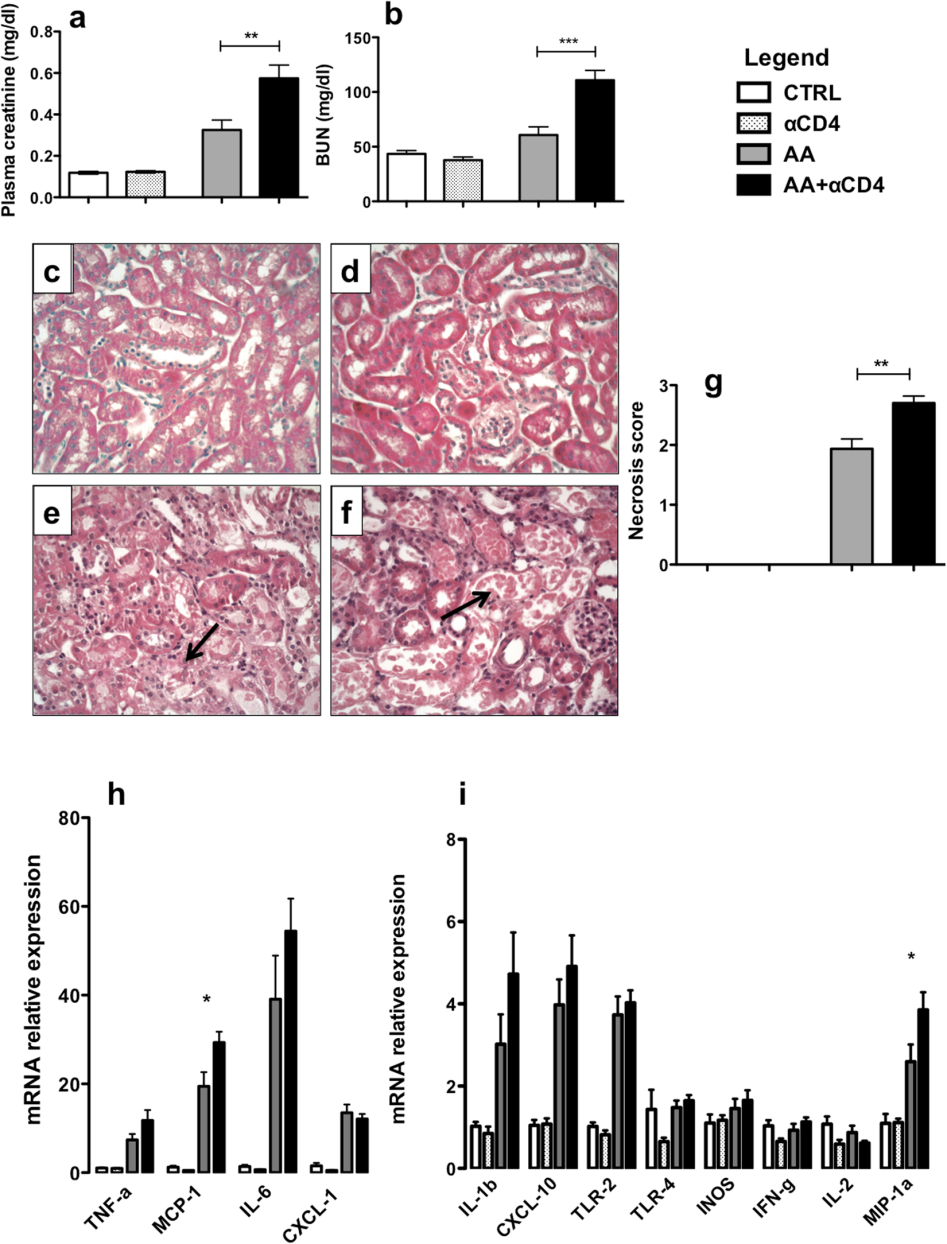
Plasma creatinine (pCr) (**a**) and blood urea nitrogen (BUN) (**b**) levels from AA + αCD4 (black columns), AA (dark greys columns), αCD4 (light greys columns) and CTRL (white columns) groups on day 5 of the protocol. A significant increase in pCr and BUN was noted in the AA + αCD4 as compared to AA group. Representative photomicrographs of renal cortex longitudinal sections in each group after 5 days of AA injection (**c**–**f**). No lesion was observed in CTRL (**c**) and αCD4 (**d**) groups. A severe tubular necrosis was observed in AA (**e**) and AA + αCD4 (**f**) groups (arrow). Original magnification x400, hematoxylin–eosin-stained kidney longitudinal sections. Histological analyses resulted in an increased necrosis score in AA + αCD4 acid as compared to AA group (**g**). Renal tissue qRT-PCR analysis of various cytokine mRNA levels on renal tissue (**h**,**i**) in the different groups. Statistical test used: Mann-Whitney. Results are expressed as the mean ± SEM, *p < 0.05; **p < 0.01; ***p < 0.001. Number of mice per group: AA + αCD4 n = 13; AA n = 14; αCD4 n = 9; CTRL n = 6.

Mice were injected for 5 days with AA and were sacrificed 24 hours after the last injection. This time point is interesting as it corresponds to the AKI phase with maximal tubular necrosis and plasma creatinine (pCr) in our model^13^. In addition, mice received anti-CD4 depleting antibody (AA + αCD4 group) or control isotype (AA group). A control group received AA vehicle and anti-CD4 (αCD4 group). Mice groups were also compared to baseline mice receiving no injection (CTRL group). A significant decrease in the percentage of CD4 T-cells was observed in the spleen (about 95%) (Supplementary Figure 1a,b) and in the kidney (more than 90%) on day 5. T-cell depletion was associated with a significant increase in pCr (p < 0.0053)) (Fig. 1a) and blood urea nitrogen (BUN) (p < 0.0004) (Fig. 1b) in the AA + αCD4 group as compared to the AA group. Next, histological lesions were assessed in each group. No lesion was observed in the CTRL or αCD4 groups (Fig. 1c,d). However, a severe tubular necrosis was observed in the AA + αCD4 and AA groups (Fig. 1e,f). Tubular necrosis score was also significantly increased in the AA + αCD4 group as compared to the AA group (p < 0.0019) (Fig. 1g). Finally, CD4^+^ T-cell depletion was associated with an increased expression of intrarenal proinflammatory mRNA cytokines: a significant increase of MCP-1 (p = 0.0240) and MIP-1α (p = 0.0289) and, as well as a non significant increase of TNF-α, IL-6, CXCL10 and IL-1β were observed (Fig. 1h,i). Renal expression of KIM-1 and NGAL mRNA were also assessed in this model. A significant increase of KIM-1 and NGAL mRNA was observed after AA injection as compared to CTRL group. However, there was no statistical difference between AA and AA + αCD4 group (Supplementary Figure 2)

In an additional set of experiments, we aimed to evaluate the impact of T-cell depletion during the chronic phase of experimental AAN. AA was injected for 5, 15 or 22 days. However, a significant mortality rate was observed in the AA + αCD4 group as compared to the AA group (p < 0.0032) (Supplementary Figure 3a). No mice died in the αCD4 group. Again, a significant increase in pCr was observed in the AA + αCD4 group as compared to the AA group after 5 days of depletion (p < 0.014). Abnormal but non-significant pCr was still observed after 15 and 22 days of injections. The pCr determination showed normal levels in the αCD4 group after 22 days of injections (Supplementary Figure 3b). Given the high mortality rate induced by AA in the depleted group, we decided to focus on day 5 of our protocol for further experiments.

### Experimental AAN is associated with an increase in regulatory T-cells in spleen and kidneys but regulatory T-cells depletion does not increase AA induced acute kidney injury

**Figure 2:**
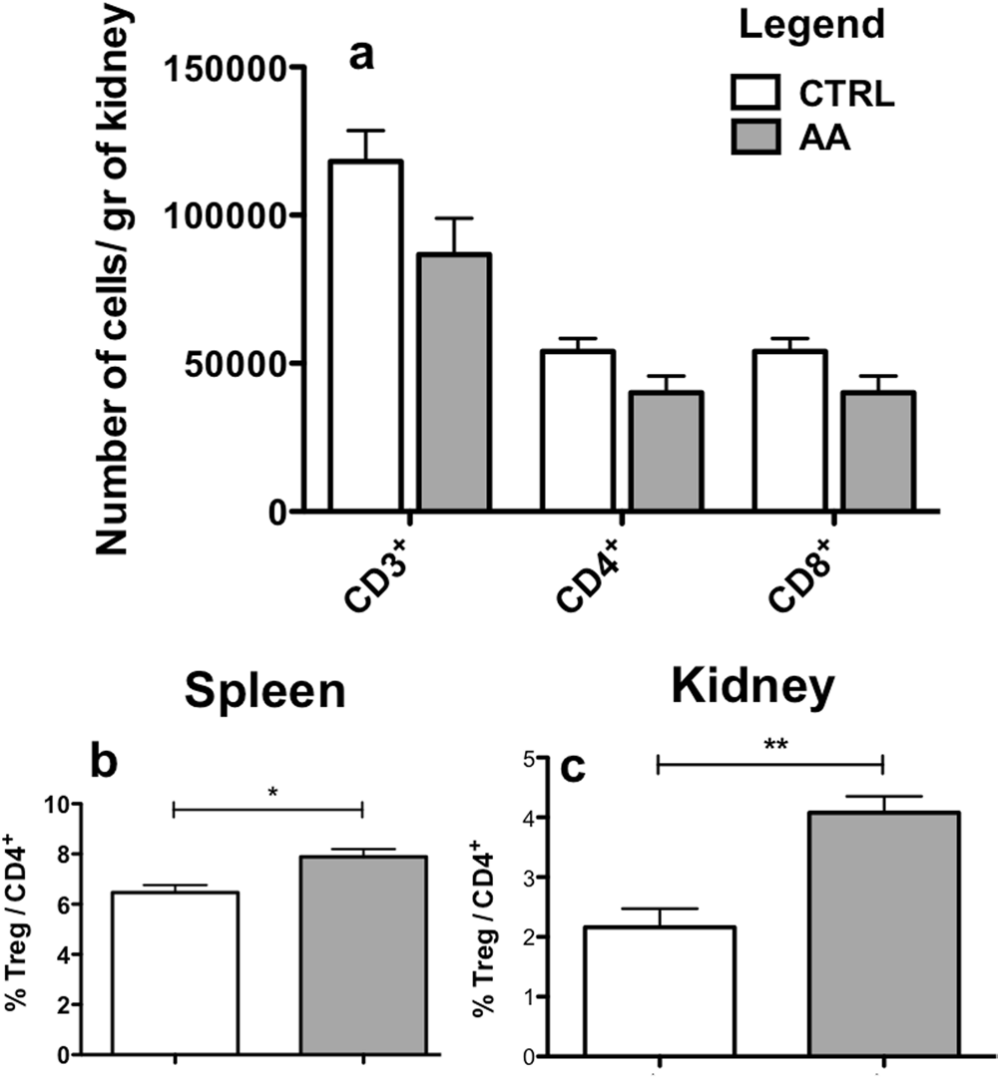
Absolute number of specific kidney lymphocyte subpopulations per gram of kidney tissue (**a**). No statistical change in the absolute number of CD3^+^, CD4^+^ or CD8^+^ lymphocytes was observed after AA injections (greys columns) as compared to CTRL group (white columns). However an increase in the percentage of splenic (**b**) and kidney (**c**) CD3^+^CD4^+^CD25^high^FOXP3^+^ T-reg cells per CD3^+^CD4^+^ T cells was observed after AA injection (greys columns) as compared to CTRL group (white columns). Statistical test used: Mann-Whitney. Results are expressed as the mean ± SEM, *p < 0.05; **p < 0.01. Number of mice per group: AA n = 14; CTRL n = 6.

Flow cytometry analyses of inflammatory kidney infiltrates after 5 days of AA injection revealed no increase in the absolute number of lymphocytic kidney (CD3^+^, CD8^+^ or CD4^+^ T-cells) infiltrates as compared to controls (p > 0.5) (Fig. 2a). However, an increase in the percentage of regulatory T-cells (T-regs) was observed in the kidneys (p = 0.0026) and spleen (p = 0.0133) of the AA group as compared to CTRL group (Fig. 2b,c). T-regs have been shown to be protective in many models of AKI such as the ischemia reperfusion injury (IRI) model^14,15^ or the toxic model^16–18^. The exacerbation of AKI in the AA + αCD4 group could be linked to the loss of T-regs. Therefore, the role of T-regs was assessed in a separate experiment.

**Figure 3:**
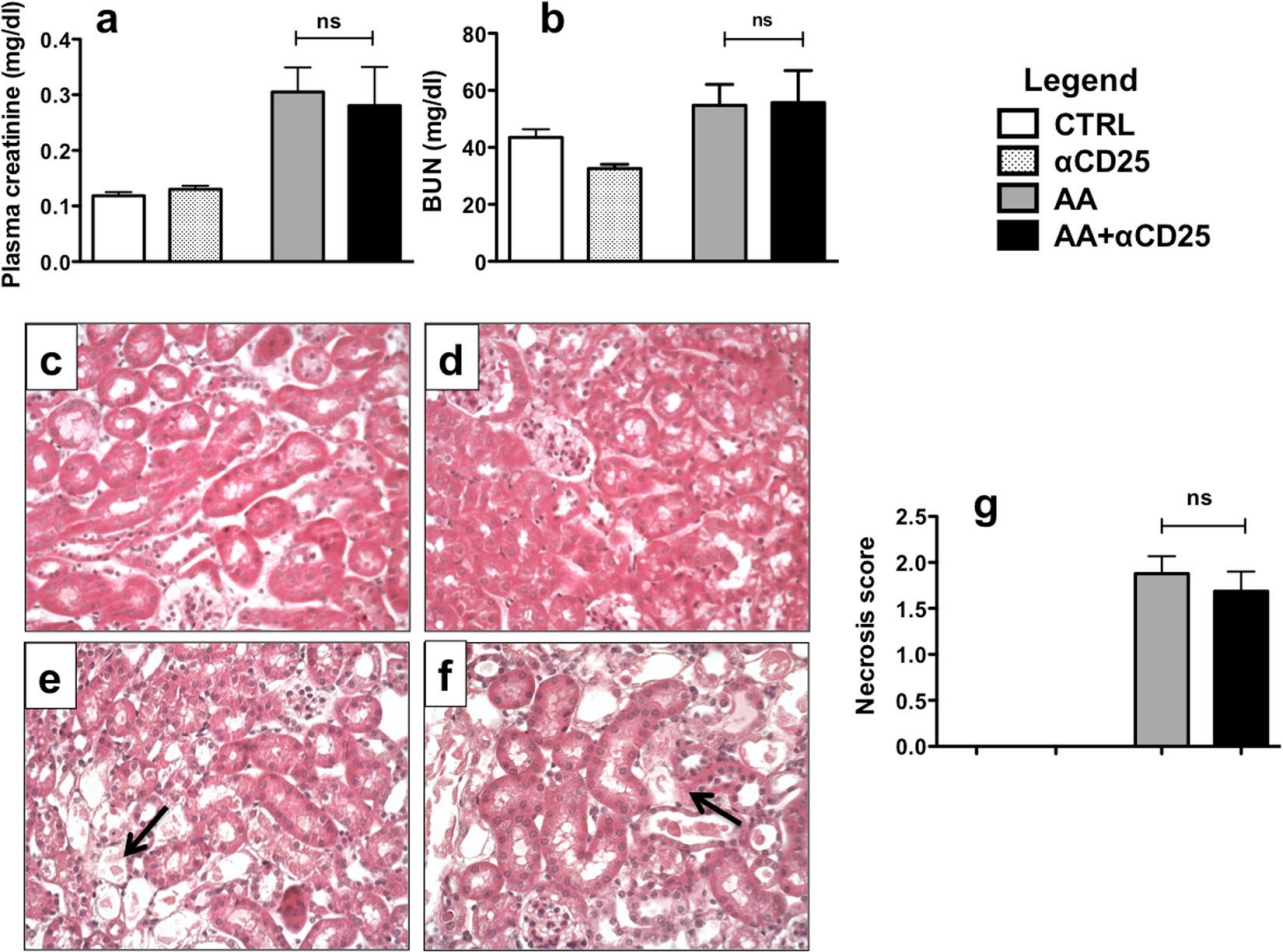
Plasma creatinine (pCr) (**a**) and blood urea nitrogen (BUN) levels (**b**) from AA + αCD25 (black columns), AA (dark greys columns), αCD25 (light greys columns) and CTRL (white columns) groups on day 5 of the protocol. There was no increase in pCr or BUN in the AA + αCD25 group as compared to AA group. Representative photomicrographs of renal cortex longitudinal sections in each group after 5 days of AA injection (**c–f**). No lesion was observed in CTRL and αCD25 groups (**c**,**d**). As with AA + αCD4, an important necrosis was observed in AA (**e**) and AA + αCD25 (**f**) groups (arrow). However necrotic lesions were not enhanced by T-regs depletion as compared to AA group. Original magnification x400, hematoxylin–eosin-stained kidney longitudinal sections. Histological analysis of necrosis injury score in the different groups (**g**). Statistical test used: Mann-Whitney. Results are expressed as the mean ± SEM, ns = no statistical difference observed. Number of mice per group: AA + αCD25 n = 7; AA n = 9; αCD25 n = 8; CTRL n = 6.

Mice were injected with anti-CD25 depleting Ab (i.e PC61) and AA (AA + αCD25 group) or with AA and control Ab (AA group). A control group was injected with AA vehicle and anti-CD25 Ab (αCD25 group). A significant decrease in the percentage of Tregs (about 75%) was observed in the spleen (Supplementary Figure 1c,d) and the kidney (more than 45%) on day 5. However, after 5 days of AA injection, Treg depletion did not result in any increase in pCr (Fig. 3a) or BUN (Fig. 3b). Tubular necrosis lesions (Fig. 3c–f) and tubular necrosis score (Fig. 3g) were almost identical to those observed in the AA group.

### CD8^+^ T-cells depletion aggravates acute kidney injury induced by AA

**Figure 4:**
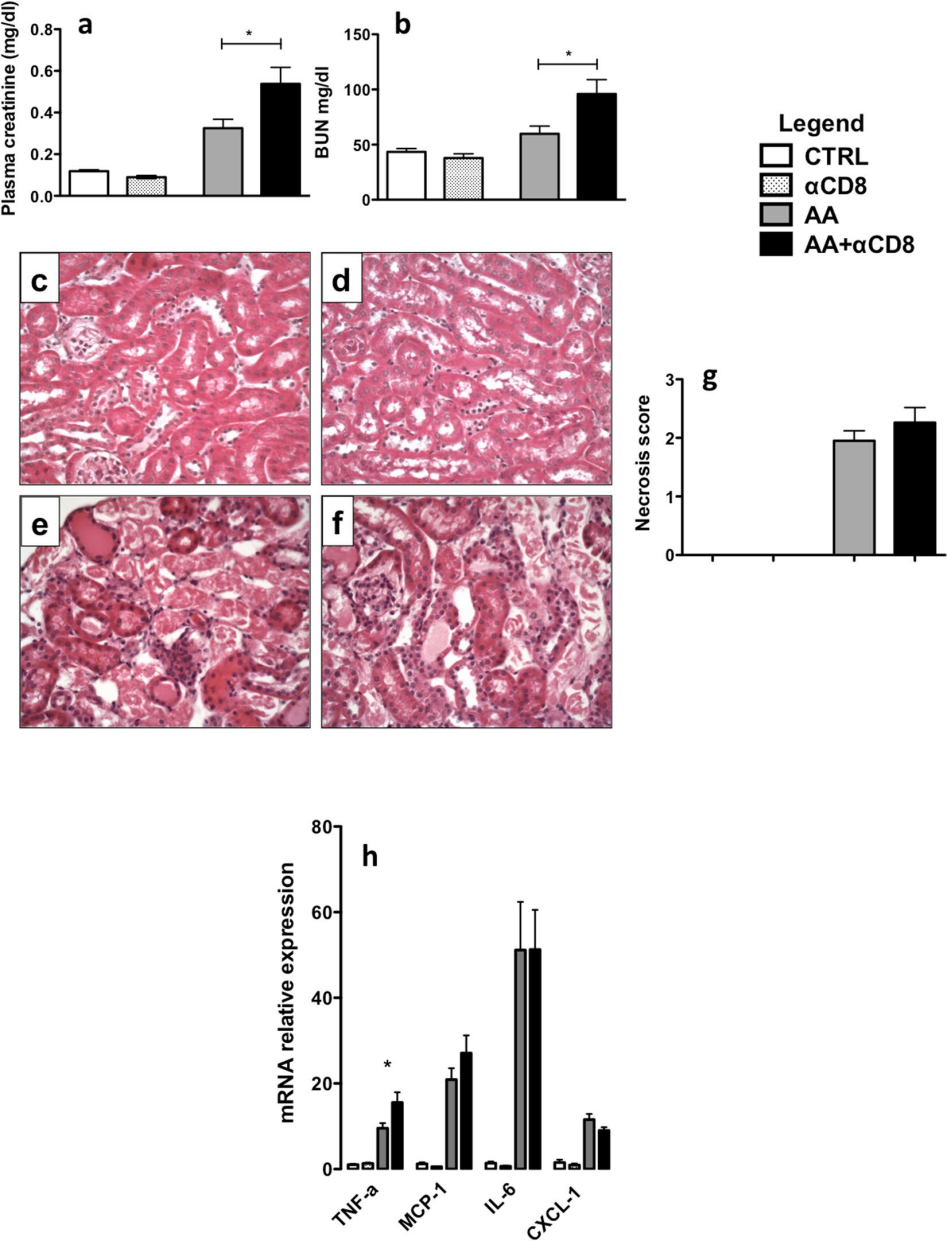
Plasma creatinine (pCr) (**a**) and blood urea nitrogen (BUN) (**b**) levels from AA + αCD8 (black columns), AA (dark greys columns), αCD8 (light greys columns) and CTRL (white columns) groups on day 5 (**a**). A significant increase in pCr and BUN was noted in the AA + αCD8 as compared to AA group. Representative photomicrographs of renal cortex longitudinal sections in each group after 5 days of AA injection (**c**–**f**). No lesion was observed in CTRL and αCD8 groups (data not shown). AA induced a massive necrosis in AA (**e**) and AA + αCD8 (**f**) groups (arrow). Original magnification x400, hematoxylin–eosin-stained kidney longitudinal sections. Histological analysis of necrosis injury score in the different groups (**g**). qRT-PCR analysis of various cytokine mRNA levels (**h**) in each group. Statistical test used: Mann-Whitney. Results are expressed as the mean ± SEM, NS = no statistical difference observed; *p < 0.05. Number of mice per group: AA + αCD8 n = 13; AA n = 14; αCD8 n = 8; CTRL n = 6.

To complete our understanding of the roles of the inflammatory infiltrate in our AAN model, we evaluated the role of CD8^+^ T-cells using depleting Ab. Mice were injected with anti-CD8^+^ YTS 169.4 and with AA (AA + αCD8 group), while another group was injected with AA vehicle and anti-CD8 (αCD8 group) or with AA and control Ab (AA group). A significant decrease in the percentage of CD8^+^ T-cells (about 90%) was observed in the spleen (Supplementary Figure 1c,d) and the kidney (more than 85%) on day 5. As with CD4^+^ T-cells depletion, worse AKI was observed as demonstrated by the significant increase in both pCr (p = 0.0415) (Fig. 4a) and BUN (p = 0.0273) (Fig. 4b). In addition, a non-significant increase in tubular necrosis and necrosis score was also observed (Fig. 4c–g). Finally, CD8^+^ T-cells depletion was associated with an increase in the expression of intrarenal pro-inflammatory cytokines, in particular a significant increase in TNF-α expression was observed (p < 0.044); in addition, MCP-1 mRNA expression tended to increase but without reaching the statistical significance (Fig. 4h).

### CD4^+^ and CD8^+^ T-cell depletions are associated with modifications in CD11b^high^F4/80^mid^ and CD11b^low^F4/80^high^ myeloid populations

**Figure 5:**
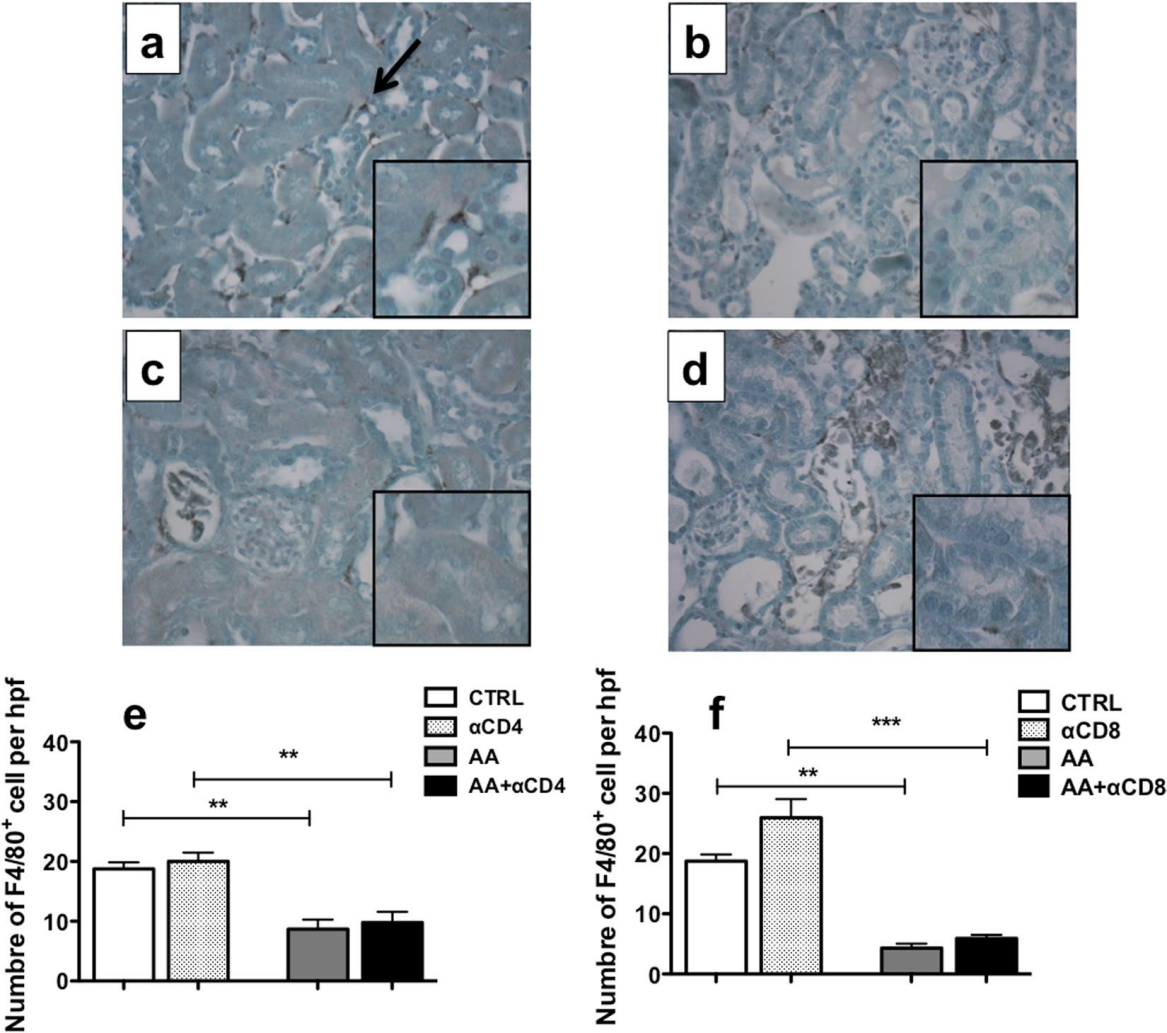
Representative photomicrographs of renal cortex longitudinal sections showing immunolocalization of F4/80 positive cells in each group (**a**–**d**). Numerous F4/80 positive cells (arrow) were localized in the interstitium in CTRL group (**a**). A drastic reduction of the number of F4/80 positive cells was observed after 5 days of AA injection in AA (**b**), AA + αCD4 (**c**) and AA + αCD8 (**d**) groups. Original magnification x400, Luxol fast blue-stained kidney longitudinal sections and magnified details showing exact position of F4/80 positive cells. The number of F4/80 positive cells per high power field (hpf) (x400) was compared in different groups (**e**,**f**). As compared to CTRL group, a significant reduction of F4/80 positive cells was observed in AA group in both experiments. However, no statistical difference was observed in the number of F4/80 positive cells between AA group and AA + αCD4 (**e**) or AA + αCD8 groups (**f**). Statistical test used: Kruskall-Wallis test followed by Mann-Whitney U-test and Bonferroni post-hoc test. Results are expressed as the mean ± SEM, ***p < 0.001; **p < 0.01; *p < 0.05. Number of mice per group: AA + αCD4 n = 13; AA n = 14; αCD4 n = 9; AA + αCD8 n = 13; αCD8 n = 8; CTRL n = 6.

AA injections did not induce changes in the absolute number of kidney lymphocytes in the early phases of the model. However, previous studies have suggested an important role for macrophages in AKI and AAN pathophysiology^19^. Therefore, we studied this population in our model. Immunohistochemistry studies revealed that F4/80 positive cells were significantly diminished after AA injections as compared to CTRL group. In addition, no difference between AA + αCD4 or AA + αCD8 and AA + CTRL groups (Fig. 5a–f) was observed.

**Figure 6:**
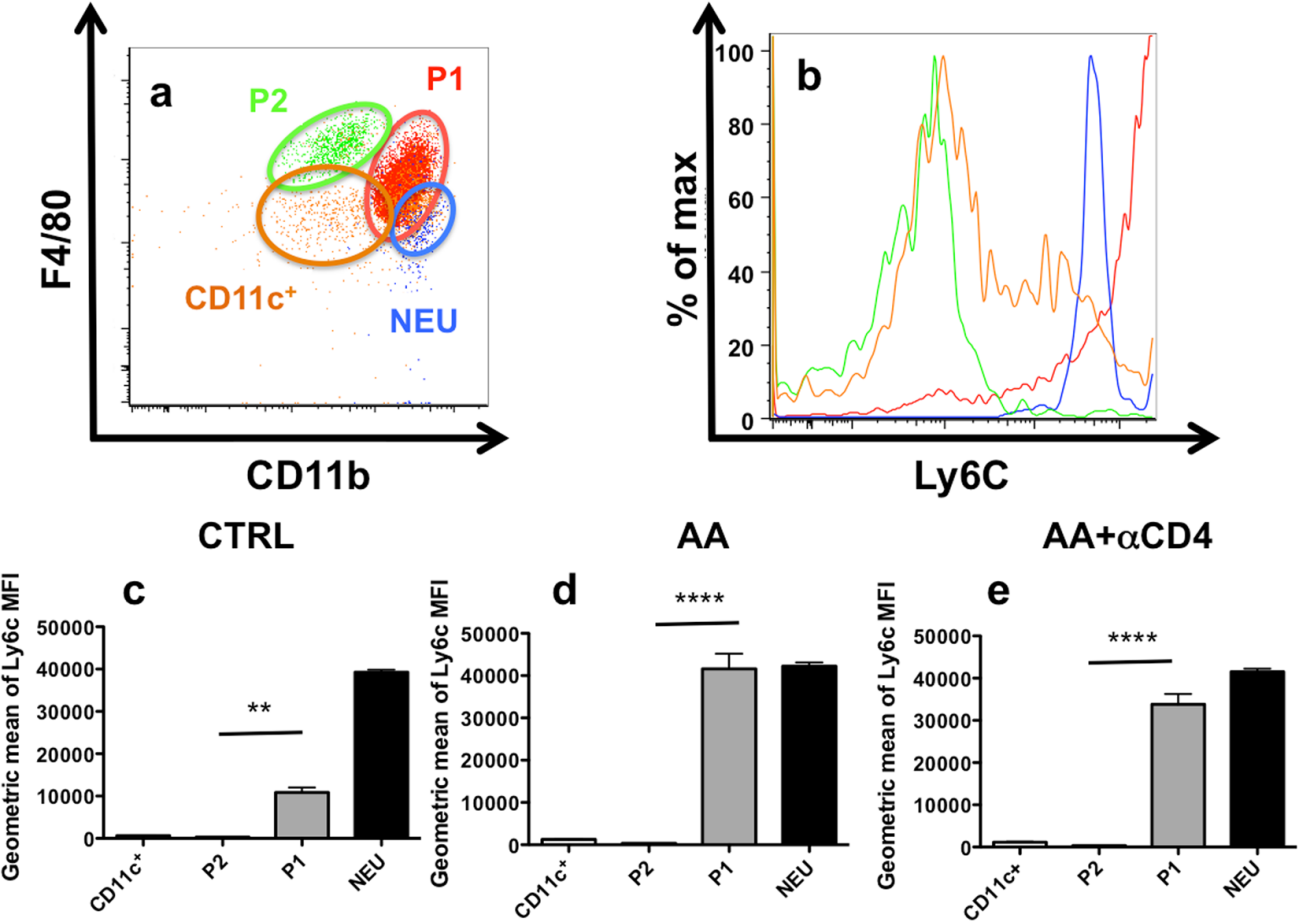
Representative dot plots showing the localization of neutrophils (NEU) (blue dots), CD11c + (orange dots), P1 (red dots) and P2 (green dots) populations on a CD11b-F4/80 diagram in a mice injected with AA (**a**). Representative histograms showing the Ly6C expression in the neutrophils (blue line), CD11c + (orange line), P2 (green line) and P1 (red line) populations in the AA group (**b**). P1 and P2 population differ from their respective Ly6C expression. Geometric mean of Ly6C MFI expression for neutrophils (black columns), CD11C + (white columns), P2 (light grey columns) and P1 populations (dark grey columns) in CTRL (**c**), AA (**d**) and AA + αCD4 (**e**) groups. A significant difference in the expression of Ly6c MFI was observed between P1 and P2 populations in different conditions. In addition, a significant increase in the geometric mean of Ly6C MFI expression in the P1 population was observed after AA injection as compared to controls (data not shown). Statistical test used: Mann-Whitney. Results are expressed as the mean ± SEM, ****p < 0.0001 and **p < 0.01. Number of mice per group: AA + αCD4 n = 13; AA n = 14; CTRL n = 6.

**Figure 7:**
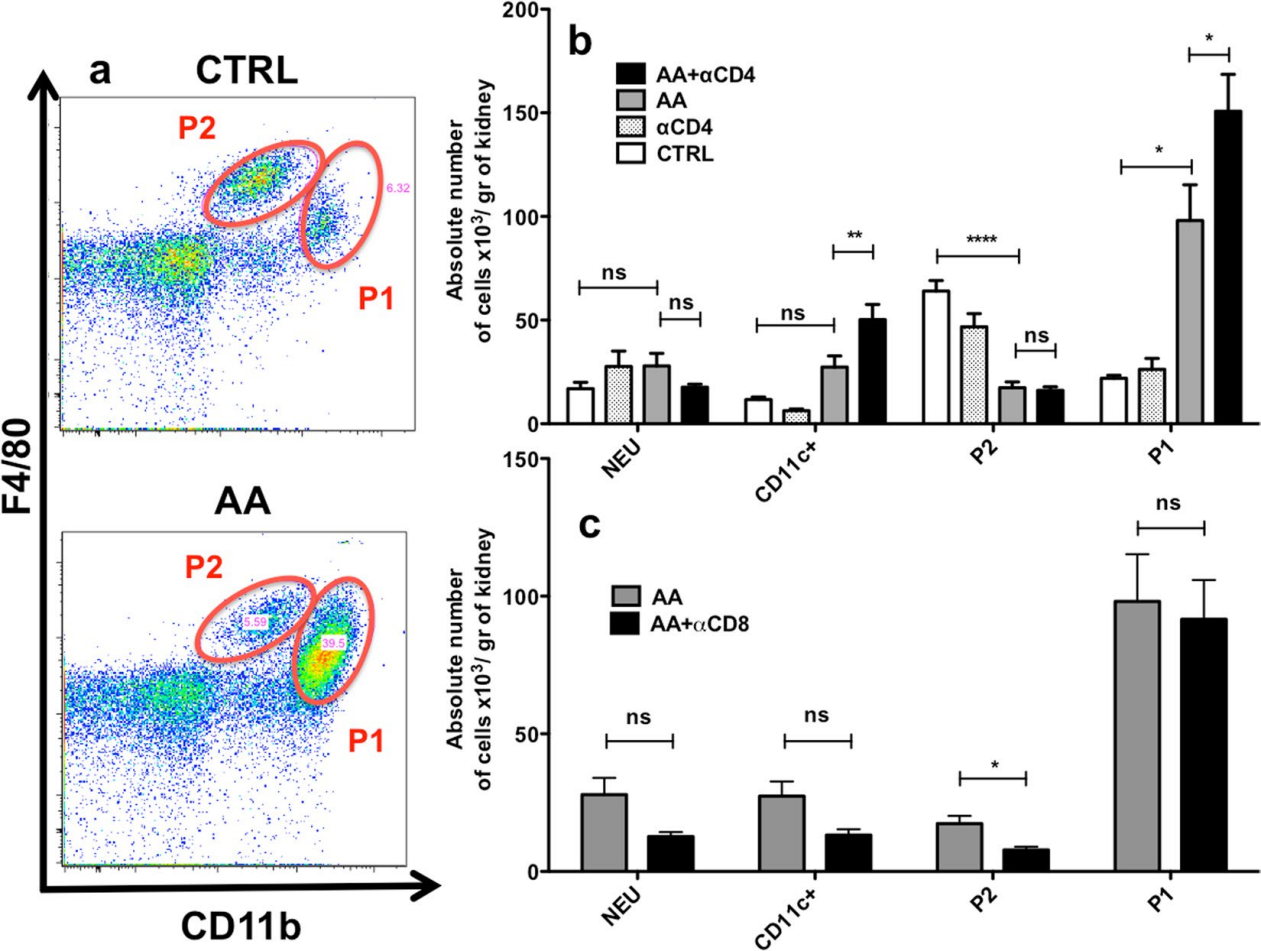
As illustrated on this representative CD11b-F4/80 dots plot, ATN lesions observed after AA injections were associated with a sharp decrease in the P2 population and a significant increase in the P1 population (**a**). Absolute number of neutrophils (NEU), CD11c+, P1 and P2 population per gram of kidney in AA + αCD4 (black columns), AA (dark greys columns), αCD4 (light greys columns) and CTRL (white columns) groups on day 5 (**b**). As compared to CTRL group, a non significant increase in the absolute number of CD11c^+^, a significant increase in the absolute number of P1 populations and a significant reduction in the number of P2 population were observed after AA injection. Moreover, in the AA + αCD4 the increase in CD11c^+^ and P1 populations was even greater than those found in AA group. After CD8 depletion, a significant decrease in P2 population was also observed as compared to AA group (**c**). Statistical test used: ANOVA followed by t-test and Bonferroni correction for multiple comparisons or Mann-Whitney U-test for simple comparisons. Results are expressed as the mean ± SEM, ****p < 0.0001, **p < 0.01, *p < 0.05; NS = no statistical difference observed. Number of mice per group: AA + αCD4 n = 13; AA n = 14; αCD4 n = 9; AA + αCD8 n = 8; αCD8 n = 8; CTRL n = 6.

As F4/80 is non-specific for macrophages and because macrophages and dendritic cells constitute heterogeneous populations with marked differences in their subpopulation, we decided to study the kidney myeloid populations more closely using flow cytometry analysis. After gating on CD45^+^ renal cells and doublets elimination, myeloid populations were separated into Ly6G^+^SSC^+^ cells (i.e neutrophils). Then, after exclusion of neutrophils (NEU), CD11c positive cells were defined based on their high expression of this marker and, finally, remaining cells were separated into CD11b^high^F4/80^mid^ (P1) and CD11b^low^F4/80^high^ population (P2) (Supplementary Figure 4). Ly6C analysis of these subpopulations revealed various expression of this marker (Fig. 6a,b). In particular, Ly6C expression profile was higher in the P1 population as compared to the P2 population in the steady state condition (Fig. 6c). Moreover, this was also the case after AA injections (AA or AA + αCD4 groups) (Fig. 6d,e), suggesting a different inflammatory profile. Finally, AA injections were associated with major modifications in the leukocytic infiltrate with an increase in the absolute number of P1 population (p < 0.05) as compared to CTRL and a decrease in the absolute number of P2 population (p < 0.0001). However, no significant modification in the absolute number of NEU or CD11c were observed (Fig. 7a,b). In addition, as compared to the AA group, the increase in CD11c^+^ (p < 0.01) and P1 (p < 0.05) populations was higher in the AA + αCD4 group (Fig. 7a,b). After AA injection and CD8^+^ T-cell depletion, there was no increase in the P1 population as compared to the AA group, however a significant decrease of P2 population was noted (p < 0.0265) (Fig. 7c).

## Discussion

AAN is recognized as a worldwide public health problem: in addition to the 1993 Belgian outbreak, when hundreds of cases of the so-called Chinese herbs nephropathy were reported, the condition has been proven to be the cause of the Balkan endemic nephropathy and of thousands of cases of CKD and cancers in Asian countries where traditional Chinese medicine is widespread^20^. AAN was firstly described as paucicellular, with scarce lymphocytic infiltrate^4^. However, this paradigm was challenged after an inflammatory infiltrate had been described in human cases and animal models^10,13,21^. Moreover, a human pilot trial with steroids has been shown to slow down the progression towards renal failure, supporting the involvement of an immune-mediated process^22^.

In our AAN model, PTEC necrosis was followed by mϕ infiltrate and by CD4^+^ and CD8^+^ T-cells influx^10^. It is important to note that the respective role of these cells has never yet been investigated before the present study. Although several other studies have demonstrated a “pathogenic” role for CD4^+^ or CD8^+^ T-cells with a less severe AKI in the absence of these cells^23–26^, we, on the other hand, surprisingly demonstrated that CD4^+^ or CD8^+^ T-cells depletion aggravated AKI after 5 days of AA injection with an increase in pCr, BUN and tubular necrotic lesions. Absence of significant difference for KIM-1 and NGAL levels between AA and AA + αCD4 group could be linked to the over-sensitivity of these markers regarding the importance of tubular necrosis observed. Moreover, when AA injections were prolonged during 22 days, the mortality rate was significantly higher in the CD4^+^ T-cells depleted group as compared to AA alone, suggesting a nephroprotective effect of CD4^+^ T-cells in long-term AA intoxication. In addition, AKI aggravation observed after CD4^+^ T-cells depletion has been found to be unrelated to Tregs subpopulation as their depletion did not increase AKI lesions in our study. Lower percentage of Treg depletion obtained in the kidney may be considered as critical in explaining the absence of any effect of their depletion. However, this magnitude of depletion is likely relevant as more than 75% of Treg were depleted in the spleen. Moreover, other authors have earlier observed a negative effect of Treg depletion in their AKI model with a similar or lower magnitude of depletion in the kidney^14,15,27,28^. Other authors have reported a protective role of CD4^+^ T-cells in a toxic injury model and in an unilateral ureteral obstruction (UUO) model^29,30^. Moreover, transfer of kidney-infiltrating lymphocytes, isolated 24 hours after renal IRI, into a T-cell-deficient mouse conferred a relative protection against subsequent ischemia reperfusion injury, suggesting a protective effect of the lymphocytes induced by renal ischemia^31^. As these cells are present in the renal interstitium in the steady state, a role in the early stage of inflammation is likely, even before the leukocyte infiltrate influx^32^.

Secondly, we described an increased P1 population infiltrate (i.e CD11b^high^F4/80^mid^) following AA injection associated with a substantial decreased P2 population (i.e CD11b^low^F4/80^high^). This observation is consistent with that of other authors in alternative AKI models^33–35^. Moreover, the increase in the P1 population was even more important in the CD4^+^ depleted mice injected with AA, while a decrease in the P2 population was observed after CD8^+^ T-cells depletion. Interestingly, Harris and colleagues suggested, in their adriamycin nephropathy model, that increase in glomerular lesions and pCr, observed after CD4 depletion, could be linked to an increase in the macrophage infiltrate (MAC-3^+^ cells)^29^. Macrophages play an essential role in AKI. After activation/injury, they can differentiate into various macrophage subtypes under the influence of their local microenvironment, affecting both their surface proteins expression and cytokines production. Basically, two well-defined phenotypes are commonly described: classically activated macrophages (M1 macrophages) and alternatively activated macrophages (M2 macrophages) with pro or anti-inflammatory properties respectively^36^. Imbalance between M1 and M2 macrophages in favour of M1 phenotype has been shown to increase AKI lesions^37,38^ whereas M2 phenotype is protective^35,38,39^. Several studies focusing on myeloid population analyses have used cytometry gating strategies similar to those used in our experimental procedures, showing that P1 had M1 macrophage characteristics and P2 had M2 macrophage characteristics^33,35^. Our results also suggest that P1 cells have a higher inflammatory phenotype as reflected by their expression of the Ly6C marker as compared to P2 cells. The restricted characterization of both P1 and P2 populations by their basic phenotype is an additional limitation. Indeed, we do not confirm that these populations are fully functional M1 and M2 macrophages as others have described^34,35,37–39^.

Finally, it was demonstrated recently that macrophages from CD4^+^ KO mice display an M1 profile, suggesting that CD4^+^ T-cells play a dominant role over other lymphocyte populations in providing the cytokine environment for regulating macrophages towards an M2 profile under normal wild-type conditions^40^. The role of the CD11c^+^ population has not been investigated in this model. However, these cells did not express high level of Ly6C,and another study had suggested a protective role of this population in toxic AKI^41^. Moreover, we demonstrated that CD4^+^ or CD8^+^ T-cell depletion in AAN was associated with renal increase in the expression of pro-inflammatory cytokines (TNF-α and MCP-1). Recent studies have suggested that adaptive immune cells were able to actively dampen initial innate responses^42^. Authors using a mouse model of TLR stimulation with poly(I:C) or LPS demonstrated that the absence of residential CD4^+^CD25^−^Foxp3^−^ or CD8^+^ T-cells resulted in a TNF-α storm produced by CD11b^+^ or CD11c^+^ cells that was responsible for an increased mortality^42^. Others have confirmed that *in vivo* T-cell–macrophage interaction through CD40 ligation with CD40L was able to down-regulate excessive TNF production by macrophages through a mechanism implicating IRAK1^43^. Taken together, our preliminary results suggest that the absence of CD4^+^ or CD8^+^ T-cells in the acute phase of our AAN model could lead to a modified inflammatory microenvironment responsible for an imbalance between M1 and M2 macrophage phenotypes and, ultimately, to more severe AKI. However, complementary investigations are necessary to confirm this hypothesis. Other physiopathological hypotheses should also be tested, such as the role of CD11c^+^ population or the role of Tr1 and gamma-delta lymphocytes, as these cells also have regulatory properties.

In conclusion, we demonstrated that CD4^+^ or CD8^+^ T-cells lymphocyte depletion was associated with AKI worsening during acute experimental AAN. This observation has been related to a modified interstitial influx in the CD11b^high^F4/80^mid^ and CD11b^low^F4/80^high^ subpopulations.

## Methods

### Experimental protocols

All protocols were approved by the Ethical Committee for Animal Care (Faculty of Medicine, Université Libre de Bruxelles). In addition, all experiments were performed in accordance with relevant guidelines and regulations. After one week of acclimatization, 10-week-old C57BL/6 male mice (Elevage Janvier, Le Genest Saint-Isle, France) were randomly assigned to one of the 7 groups (Supplementary Figure 5). Depending on their groups, mice were injected with AA + anti-CD4 Ab (GK1.5; Bioxcell, West Lebanon, NH USA) (AA + αCD4 group); with AA + anti-CD8 Ab (YTS 169.4; Bioxcell) (AA + αCD8 group); with AA + control isotype (LTF-2; Bioxcell) (AA group); with AA + anti-CD25 Ab (PC61; Bioxcell) (AA + αCD25 group); with AA vehicle (i.e polyethylene-glycol, PEG) (Fluka Chemie, Buchs, Switzerland) + anti-CD4 Ab (αCD4 group); with PEG + anti-CD8 Ab (αCD8 group); or PEG + anti-25 Ab (αCD25 group). In addition, a control group did not receive any injection and was used as baseline day 0 (CTRL). AA (5mgr/kg body weight) or an equivalent volume of PEG was administered by means of daily IP injections during five days and GK1.5 or YTS169.4 (0.250 mg) or control isotype (0.250 mg) were administered by means of a single IP injections 72 hours before the first injection of AA. PC61 (0.300 mg) was injected twice, i.e. 72 and 24 hours before the first AA injection. AA (Acros Organics Co., Geel, Belgium; 40% AAI, 60% AAII,) was given in 150 µl of PEG and sterile water. GK1.5, YTS 169.4 and LTF-2 were given in 150 µl of sterile dPBS. For the chronic phase experiment, mice were injected with AA for 5, 15 or 22 days as described above. Ab injections were repeated weekly until sacrifice. CD4^+^ T-cell depletion (>95%) was effective during all protocol (supplementary Figure 1a,b). Twenty four hours after the last AA injection, mice were anesthetised with ketamine-HCl (Merial, Brussels, Belgium) and 2% xylazine (Bayer, Brussels, Belgium) after which a blood specimen was obtained by cardiac puncture. Then, kidneys were flushed with 30 ml of NaCl 0,9% and kidneys were harvested for analysis. Left kidneys were used for flow cytometric analysis whereas right kidneys were used for realtime polymerase chain reaction (rtPCR) (immediately snap frozen in liquid nitrogen and stored at −80 °C) and for histological analysis.

### Renal histopathology

4% buffered formaldehyde fixed and paraffin embedded (FFPE)) sections (4 μm) were attached to poly-L-lysine pre-treated slides (Sigma-Aldrich, Bornem, Belgium). After air-drying, the paraffin from tissue sections was removed (xylene solution). Tubular damages were assessed after hematoxylin/eosin coloration by scoring tubular necrosis in 10 non-overlapping fields (400× magnification) in the cortex and corticomedullary junction. Injury was scored by a pathologist (TB) blinded for the groups on a 5-point semi-quantitative score: 0 = no damage, 1 less than 10% of the cortex and corticomedullary junction injured, 2 = 11–25%, 3 = 26–50%, 4 = 51–75%, 5 more than 75% of the cortex and corticomedullary junction injured.

### Immunohistochemistry

PBS was used for all washing steps. The FFPE sections (4 μm) were deparaffined, rehydrated and immersed in a retrieval sodium citrate buffer (pH 6.0) solution to unmask antigen. Endogenous peroxidase activity was quenched with 0.3% hydrogen peroxide in a methanol solution (30 min). After blocking non-specific site, tissue sections were incubated overnight with primary antibodies (rat anti-mouse Ab F4/80, clone RM0029–11H3, Abcam, UK). Then, slides were exposed for 30 min to the secondary Ab, kidney sections were finally incubated with strepta-HRP complex for 30 min, and bound peroxidase activity was detected with the DAB kit (Dako, Heverlee, Belgium). Counterstaining with Luxaol Fast blue completed the processing. The specificity of Ab used was established by the producer. Normal serum (5% solution) instead of the primary Ab (used in order to exclude non-specific staining of kit reagents) showed no staining.

### Quantification of immunostainings

Quantifications were performed by two investigators (TB and IJ) who were blinded to the experimental groups. F4/80 positive cells were counted on ten photographs of non-overlapping 400× field, after which mean for the two investigators was calculated for each photograph.

### Biochemical evaluation of renal function

Plasma creatinine (Pcr) excretion levels were determined as previously described using an HPLC technique. Plasma BUN were determined using Quantichrom Urea Assay Kit (Bioassay systems, Gentaur, Brussels, Belgium).

### Isolation of lymphocytes from mouse kidneys

Kidney leukocytes were isolated using a technique described previously, with few modifications^32^. In short, organs were disrupted mechanically in 5 ml RPMI 1640 medium (PAA, Pasching, Austria)+ 10% FBS using a syringe plunger in a petri dish. To remove debris, samples were passed through a 40 micrometre filter that was then rinsed with 15 ml of RPMI 1640 medium. Next, cells were suspended in 5 ml of 36% Percoll (Sigma, Belgium Overijse), gently overlaid onto 72% Percoll and centrifuged at 1000 *g* for 25 min at room temperature. Cells were isolated from the Percoll interface and washed in medium at 300 *g* for 5 min at 4 °C, after which they were resuspended in 2 ml of RPMI and finally divided in two equal parts for immunostaining.

### Antibodies

The fluorochrome-conjugated mAb to mouse antigens used for flow cytometry analysis were from eBioscience (VWR, Haasrode, Belgium): anti-CD16/CD32, anti-Ly-6c (PE conjugated; clone HK1.4), anti F4/80 (APC conjugated; clone BM8), anti-Foxp3 (APC conjugated; clone FJK-16s); from Beckton Dickinson (Erembodegem, Belgium): anti-CD3 (PerCp conjugated; clone 145-2C11), anti-CD4 (FITC conjugated; clone RMA4-4), anti-CD8 (Pe-Cy7 conjugated; clone 53-6.7), anti-CD45 (APC-Cy7 conjugated; clone 30-F11), anti-CD25 (PE conjugated; clone 7D4), anti-CD11b (FITC conjugated; clone M1/70); or from Biolegend (Imtec, Antwerpen Belgium): anti-CD11c (Pe/Cy7 conjugated; clone N418), anti-Ly-6G (Brillant violet 421 conjugated; clone 1A8). GK1.5 mAb reportedly do not block binding of RM4-4 antibodies.

### Flow cytometry analysis

Lymphocytes were preincubated with anti-CD16/CD32 for 30 min to minimize nonspecific Ab binding. Cells were then incubated with various combinations of mAb for a minimum of 1 hour at 4 °C. One half was stained with anti-CD45, CD3, CD4, CD8 and CD25. After fixation and permeabilization with Fixpem solution (eBioscience) for 1 hour at 4 °C, cells were finally incubated with anti-Foxp3 Ab. The second half was stained with anti-CD45, CD11b, CD11c, Ly-6G, Ly6C and F4/80. Eight-colours immunofluorescence staining was analyzed using a FACSCanto II instrument (BD Biosciences). The leukocytes were gated using CD45 and side-scatter (SSC) followed by exclusion of debris, dead cells and doublets. Gating strategies for myeloid population are summarized in supplementary Figure 4. T-regs were defined as CD45^+^CD3^+^CD4^+^CD25^high^FOXP3^+^ cells.

### Cell count

The absolute number of kidney lymphocytes was determined using the Trucount (Beckton Dickinson) tubes following the instructions from the manufacturer. The absolute number of cells was expressed as number of cells per gram of kidneys (cell/organ) excepting T-regs which are a percentage of CD4^+^ T-cells.

### Messenger RNA quantification via Real-Time Reverse-transcriptase polymerase chain reaction

Frozen kidney samples were homogenized in a lysis solution with a MagNalyser (Roche Diagnostics, Brussels, Belgium). Total RNA was then extracted with the High Pure RNA Tissue Kit (Roche Diagnostics) according to the manufacturer’s protocol, which included DNase treatment. The mRNA quantification was performed using a 2-step real-time reverse-transcriptase polymerase chain reaction (LightCycler, Roche Diagnostics). The primers and probes were designed using Primer3 software (Whitehead Institute for Biomedical Research, Cambridge, MA) or were purchased as ready to use (Life technologies, Ghent Belgium) (Table 1). We used HPRT as the housekeeping gene. Data are expressed using the comparative threshold cycle (dCt) method, and mRNA ratios are given by 2^−dCT^ as compared to CTRL group.

**Table 1:** ABS: applied biosystem; EG: Eurogentec; NA: non available.

Protein	Supplier	Forward, 5′ -3′	Reverse, 5′ -3′	#
IL-1b	ABS	na	na	Mm00434228_m1
IL-2	EG	CAGGATGCTCACCTTCAAATT	CAAGTTCATCTTCTAGGCACTGAA	na
CXCL10	ABS	na	na	Mm00445235_m1
MCP-1	EG	GCTCAGCCAGATGCAGTTAAC	CCTACTCATTGGGATCATCTTG	na
IFNγ	EG	GGATGCATTCATGAGTATTGC	GCTTCCTGAGGCTGGATTC	na
TNFα	EG	CAGACCCTCACACTCAGATCA	CACTTGGTGGTTTGCTACGA	na
HPRT	EG	GGACCTCTCGAAGTGTTGGAT	CCAACAACAAACTTGTCTGGAA	na
IL-6	ABS	na	na	Mm00446190_m1
CXCL1	ABS	na	na	Mm04207460_m1
TLR-2	EG	CCTACATTGGCCATGGTGAC	CCTCTATTGTATTGATTCTGCTGGA	na
TLR-4	EG	TGTTGCTTGTATATGTGAACATCAG	TCAACATTCACCAAGAACTGC	na
INOS	EG	CAGCTGGGCTGTACAAACCTT	CATTGGAAGTGAAGCGTTTCG	na
MIP1-α	EG	AAGTCTTCTCAGCGCCATATG	GTGGAATCTTCCGGCTGTAG	na

### Statistical analysis

Wilk-Shapiro test and Q-Q plots are used to verify if there is any deviation from the normality for the continuous variables. For group comparisons, Mann-Whitney tests, Kruskall-Wallis test followed by Mann-Whitney U-test or One Way ANOVA followed by t-test were used when appropriate. The Bonferroni correction has been used for multiple comparisons. For survival study, Log-rank (Mantel-Cox) test was used. All analyses were performed using Prism 5.0c software for Macintosh (GraphPad Software, San Diego California USA) or SPSS.v24 (SPSS Inc Chicago, Illinois, USA).

### Data Availability

The datasets generated during and/or analyzed during the current study are available from the corresponding author on reasonable request.

## Electronic supplementary material

Supplementary data
