# Supplementary material for: CD4+ and CD8+ T Cells Exert Regulatory Properties During Experimental Acute Aristolochic Acid Nephropathy
Source: Sci Rep. 2018 Mar 28;8:5334. doi: 10.1038/s41598-018-23565-2 (PMC5871862; doi:10.1038/s41598-018-23565-2)
Supplement: Supplementary file 1 — Supplementary data [file 41598_2018_23565_MOESM1_ESM.docx]

CD4+ AND CD8+ T CELLS EXERT REGULATORY PROPERTIES DURING EXPERIMENTAL ACUTE ARISTOLOCHIC ACID NEPHROPATHY

**Thomas Baudoux, Cécile Husson^1^, Eric De Prez, Inès Jadot, Marie-Hélène Antoine, Joëlle L. Nortier and Jean-Michel Hougardy**

**Supplementary Figure 1**


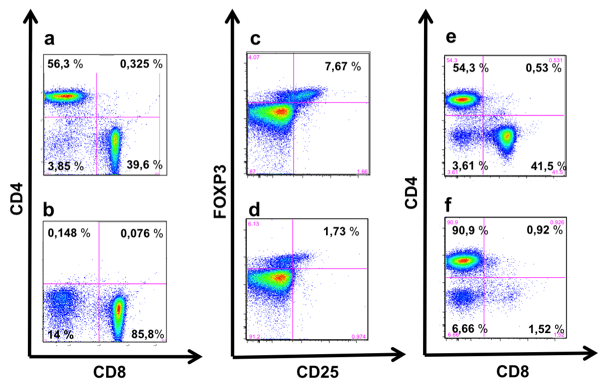


Representative cytometry spleen dot plots showing the depletion of CD4^+^(**b**), CD8^+^(**d**) or T-reg (**f**) lymphocytes after corresponding antibodies injections as compared to AA groups (**a,c,e**).

**Supplementary Figure 2**

**
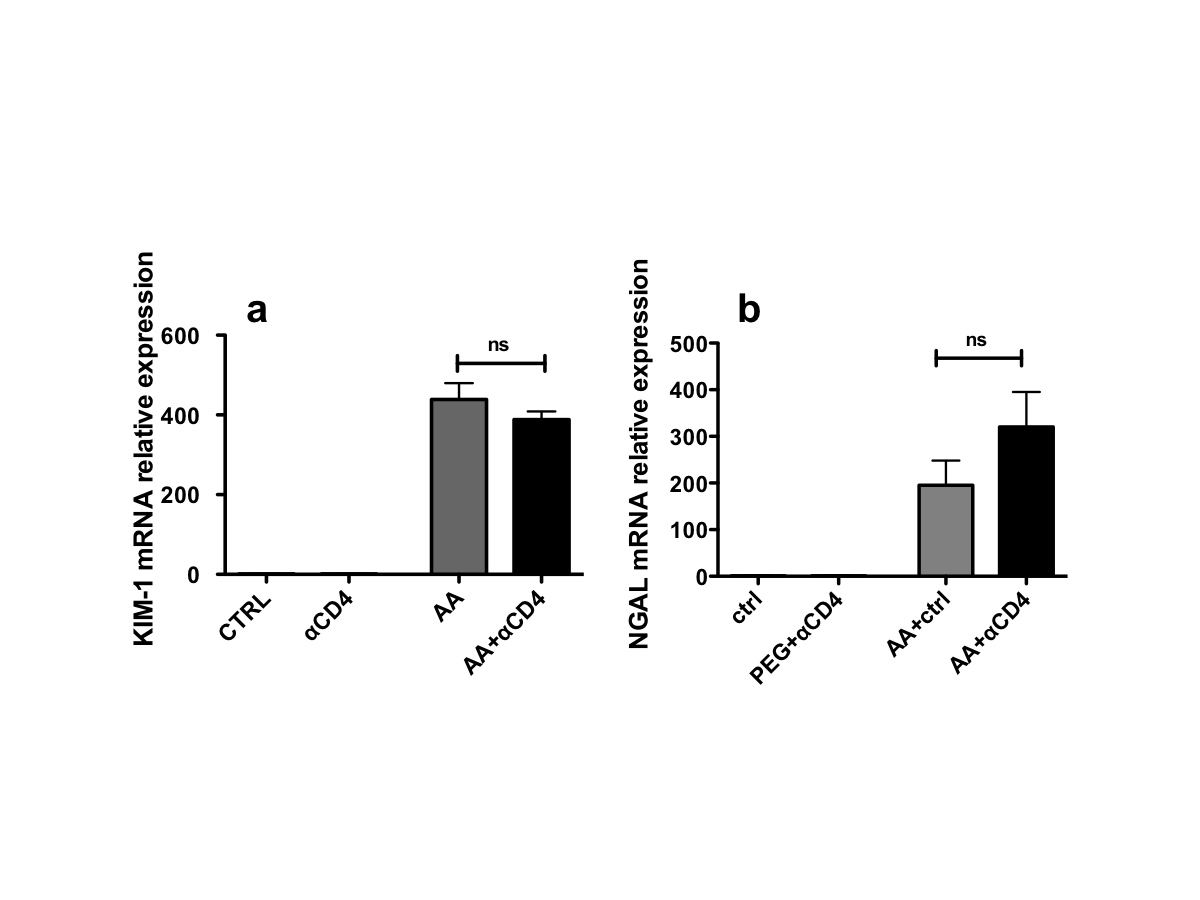
**

Renal tissue qRT-PCR analysis of KIM-1 (a) and NGAL (b) mRNA levels on renal tissue in different mice groups during the acute phase model (time point at day 5). Results are expressed as the mean ± SEM; ns = no statistical difference observed. (AA+αCD4 n =13 ; AA n = 14 ; αCD4 n =9 ; CTRL n= 6).

**Supplementary Figure 3**


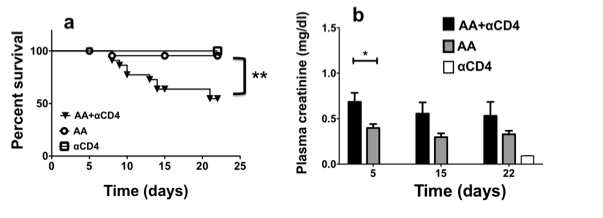


Kaplan-Meier survival percentages **(a)** in AA+αCD4 (black triangles), AA (white circles) and αCD4 (white squares) groups**.** A significant survival difference between AA+αCD4 and AA was observed (Log-rank Mantel-Cox) test. No mortality was observed in αCD4 group. Plasma creatinine (pCr) levels from AA+αCD4 (black columns), AA (greys columns) and αCD4 (white columns) groups on days 5, 15 and 22 **(b)**. An increase in pCr was noted after CD4 T-cells depletion as compared to AA group. Statistical test used: Mann-Whitney and Log-rank (Mantel-Cox). Results are expressed as the mean ± SEM, ★p < 0.05; ★★p < 0.01. Number of mice per group : AA+αCD4 day5 n = 7 ; AA+αCD4 day15 n = 6; AA+αCD4 day22 n = 6 ; AA day5 n = 8 ; AA day15 n = 9; AA day22 n = 12 ; αCD4 day22 n = 8).

**Supplementary Figure 4**


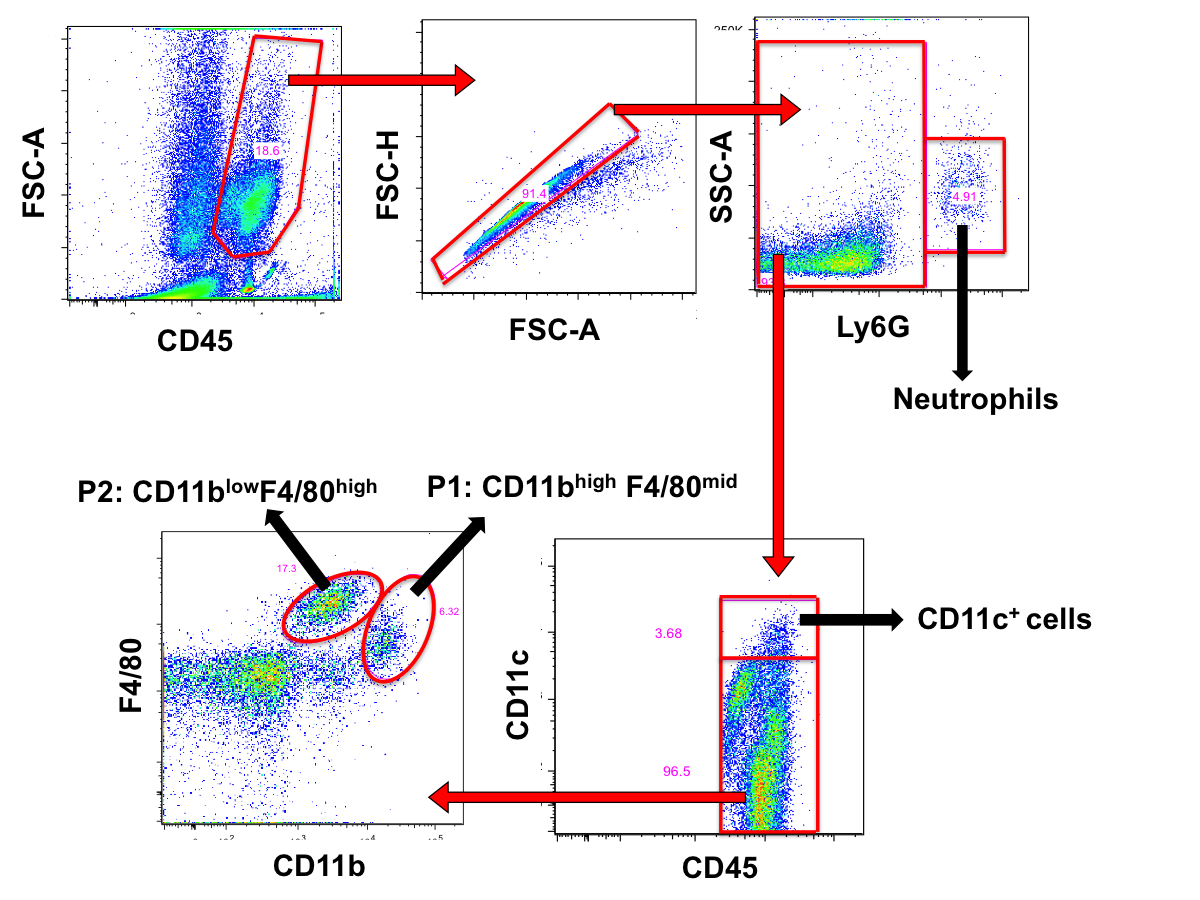


Representative dot plots showing the gating strategy for multicolour flow cytometry analyses of specific kidney myeloid populations. After gating on CD45^+^ cells, doublets were eliminated. Then, neutrophils were defined as Ly6G^+^ and SSC^+^ cells. Within the remaining cells, CD11c^high^ positive cells were defined as CD11c^+^, then P1 and P2 population were separated based on CD11b and F4/80 markers.

**Supplementary Figure 5**

**
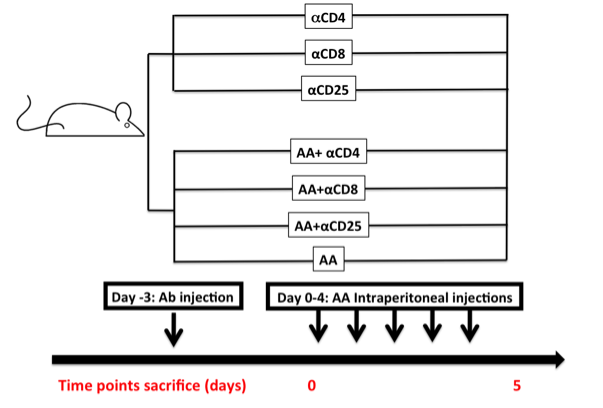
**

Schematic representation of experimental protocols performed in the mouse model of acute aristolochic acid nephropathy (AAN). Ten weeks old C57BL/6 male mice were randomized in the different groups (n=6-14 per group). Aristolochic acid (AA) (5 mg/kg body weight) or AA vehicle (PEG) was injected once a day I.P and antibodies (i.e anti-CD4: 250 μgr of GK1.5, anti-CD8: 250 μgr of YTS169.4 or control isotype: 250 μgr of LTF-2) were injected once 72 hours before the first AA/PEG injection. Anti-CD25: 300 μgr of PC61 were injected twice 72 and 24 hours before the first AA injection. After 5 days of AA injection mice were sacrificed and blood sample and kidneys were harvested for further analysis. One group of mice receiving no injection was used as baseline.
